# MRI-Based Vertebral Bone Quality Score for QCT-Defined Osteoporosis: Incremental Diagnostic Value and an Exploratory 1.5-T Threshold in a Retrospective Study

**DOI:** 10.3390/jcm15218752

**Published:** 2026-11-09

**Authors:** Zhongzheng Yan, Gaohai Shao, Meichao Deng, Xun Tang, Mengfan Yang, Yiming Qu, Hongjun Zhang

**Affiliations:** Affiliated Yongchuan Hospital of Chongqing Medical University, No. 439 Xuanhua Road, Yongchuan District, Chongqing 402160, China; yanzhonzheng123@163.com (Z.Y.);

**Keywords:** osteoporosis, magnetic resonance imaging, vertebral bone quality, quantitative computed tomography, volumetric bone mineral density, fat fraction, internal validation, opportunistic case finding

## Abstract

**Background:** Osteoporosis is frequently underrecognized in patients undergoing lumbar magnetic resonance imaging (MRI), creating an opportunity for imaging-based case finding. **Methods:** This retrospective diagnostic accuracy study evaluated the MRI-based vertebral bone quality (VBQ) score against quantitative computed tomography (QCT)-defined osteoporosis, its incremental diagnostic value beyond age and sex, and the stability of a threshold derived under a standardized 1.5-T protocol. Among 281 consecutive adults, 278 formed the primary MRI cohort and 234 with valid L1–L2 QCT measurements formed the reference-standard cohort; 51 had osteoporosis. VBQ was calculated from L1–L4 T1-weighted vertebral signal intensities normalized to L3 cerebrospinal fluid intensity. **Results:** Interobserver reliability was high (intraclass correlation coefficient (ICC(2,2)), 0.919; 95% confidence interval (CI), 0.899–0.935). VBQ correlated inversely with QCT volumetric bone mineral density (r = −0.687; *p* < 0.001) and remained independently associated with osteoporosis after adjustment for age and sex (adjusted OR, 6.08; 95% CI, 2.36–15.68). Adding VBQ increased the apparent model area under the receiver operating characteristic curve (AUC) from 0.847 to 0.896 (bootstrap optimism-corrected AUCs, 0.839 and 0.887). Standalone AUCs were 0.833 for VBQ and 0.863 for fat fraction. A VBQ threshold >3.7047 yielded 74.51% sensitivity and 83.06% specificity, but bootstrap-selected thresholds varied (2.5th–97.5th percentiles, 3.409–3.851). **Conclusion:** VBQ may provide an adjunctive opportunistic marker on routine lumbar MRI. However, its threshold is exploratory and requires external validation; VBQ should not be used alone to exclude osteoporosis.

## 1. Introduction

Osteoporosis is a systemic skeletal disorder characterized by reduced bone mass and deterioration of bone microarchitecture, resulting in increased bone fragility and fracture risk [1]. It remains a major global health problem, with substantial fracture-related morbidity and disability [2,3,4]. Patients undergoing lumbar imaging for low back pain or other spinal complaints may have unrecognized low bone density, creating an opportunity for opportunistic case finding during examinations performed for other clinical indications.

Dual-energy X-ray absorptiometry (DXA) remains a widely used modality for clinical bone mineral density (BMD) assessment [5]. However, lumbar DXA measurements may be spuriously elevated by osteophytes, endplate sclerosis, and other degenerative changes [6,7]. Quantitative computed tomography (QCT) provides three-dimensional measurements of trabecular volumetric BMD (vBMD) and is less susceptible to some of these confounding effects, although radiation exposure, cost, calibration requirements, and dedicated analysis limit its use for routine screening [8,9]. An MRI-derived marker obtained from an existing lumbar examination could therefore provide complementary information without an additional imaging procedure.

Bone loss is frequently accompanied by increasing vertebral marrow adiposity, a biological change that may be reflected in T1-weighted MRI signal [10,11,12,13,14]. The MRI-based vertebral bone quality (VBQ) score has emerged as a potential opportunistic marker of bone health on routine lumbar MRI [15,16,17]. It is derived from the ratio of vertebral marrow signal intensity to cerebrospinal fluid (CSF) signal intensity and may reflect marrow-composition changes associated with reduced bone density. Previous investigations have examined VBQ in patients with osteoporosis, fragility fractures, and spinal disorders [18,19,20,21,22,23].

Comparisons of VBQ with DXA or QCT, investigations across magnetic field strengths, and reproducibility studies have reported variable diagnostic thresholds [24,25,26,27], although meta-analyses support its potential for identifying low bone density [28,29]. Such variation may reflect differences in field strength, acquisition parameters, region-of-interest (ROI) placement, study populations, reference standards, and cutoff selection. Moreover, thresholds derived and evaluated in the same cohort may overestimate diagnostic performance. The present study therefore evaluated associations between VBQ and QCT vBMD under a standardized 1.5-T protocol, assessed reader reliability, determined whether VBQ added diagnostic information beyond age and sex, compared VBQ with fat fraction (FF) and R2*, and explored the stability of a data-derived cutoff.

## 2. Materials and Methods

### 2.1. Study Design and Participants

This single-center retrospective diagnostic accuracy study identified 281 consecutive adults who underwent lumbar MRI for low back pain or other clinical spinal complaints at the Affiliated Yongchuan Hospital of Chongqing Medical University between January 2024 and August 2025. Written research consent had been obtained during the patients’ clinical visits. The study was reviewed and approved for retrospective analysis by the hospital ethics committee (approval no. 2025EC0301; review date, 12 December 2025). For participants with QCT data, the maximum interval between lumbar MRI and QCT was 7 days. Eligibility required age ≥18 years, evaluable L1–L4 T1-weighted images, and a suitable CSF reference measurement. Previous lumbar surgery or instrumentation, malignancy, metabolic bone disease other than osteoporosis, long-term treatment substantially affecting bone metabolism, and inadequate image quality were exclusion criteria.

**Figure 1:**
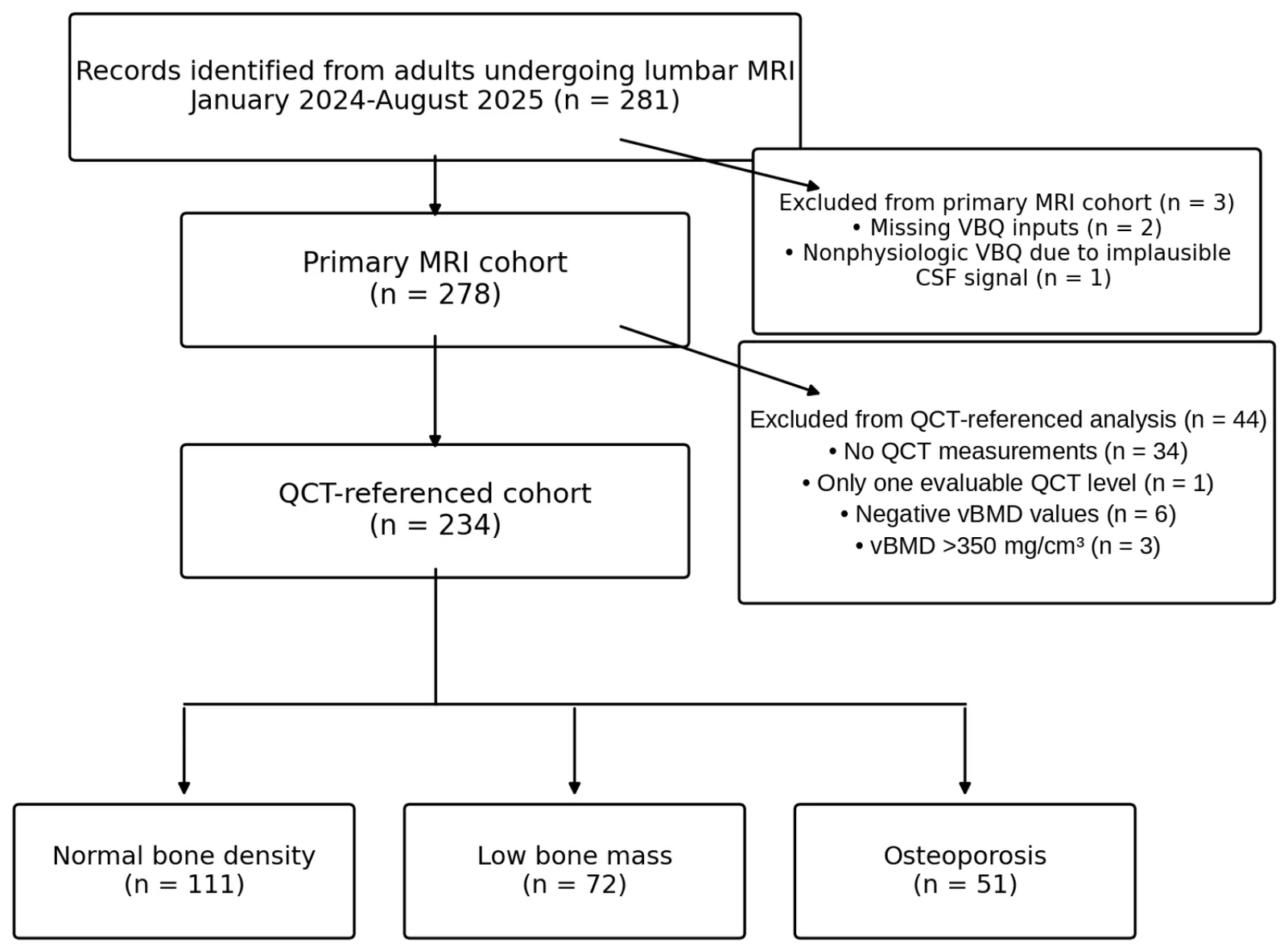
Flow of consecutively identified patients (*n* = 281) into the MRI cohort (*n* = 278) and QCT-referenced diagnostic accuracy cohort (*n* = 234). Thirty-four MRI participants had not undergone QCT; ten had incomplete or potentially invalid QCT values. CSF, cerebrospinal fluid; VBQ, vertebral bone quality; QCT, quantitative computed tomography; vBMD, volumetric BMD.

Two records lacked the measurements required for VBQ calculation, and one record contained a grossly implausible CSF-normalized VBQ value; these records were excluded from the primary MRI cohort, leaving 278 participants. QCT-referenced analyses additionally required valid L1 and L2 measurements. Among the 278 MRI-eligible participants, 34 had no available QCT measurements, one had only one evaluable QCT level, six had a negative vBMD measurement at one level, and three had at least one measurement >350 mg/cm^3^. These 44 participants were excluded from the primary QCT-referenced analysis, leaving 234 participants. In the three records with measurements >350 mg/cm^3^, two had concordant values of 425 mg/cm^3^ at both measured levels, whereas the third had markedly discrepant values. The implications of incomplete verification and the exclusion of abnormal measurements were considered when interpreting diagnostic performance (Figure 1).

For descriptive subgroup analyses, age was prespecified as 18–44 years (younger adults), 45–59 years (middle-aged adults), and ≥60 years (older adults). The ≥60-year cutoff followed the United Nations convention for defining older persons [30]; the two younger strata were study-defined categories selected to distinguish early and middle adulthood. Age was retained as a continuous variable in the primary multivariable model.

### 2.2. Imaging Acquisition and Quantitative Measurements

All participants underwent lumbar MRI using the same 1.5-T scanner (uMR 588; United Imaging Healthcare, Shanghai, China) and a standardized examination protocol. The noncontrast sagittal T1-weighted fast spin-echo sequence used for VBQ measurement had a repetition time (TR) of 599 ms, echo time (TE) of 8.44 ms, slice thickness of 4 mm, interslice gap of 0.4 mm, reconstructed matrix of 456 × 456, and reconstructed field of view of approximately 320 × 320 mm. Routine sagittal T2-weighted imaging used a TR/TE of 2526/105.6 ms, slice thickness of 4 mm, and interslice gap of 0.4 mm. Sagittal quantitative FF and R2* maps were obtained using a FACT3d three-dimensional gradient-echo sequence (TR, 11.13 ms; flip angle, 3°; thickness, 4 mm; reconstructed matrix, 256 × 200; pixel size, 1.25 × 1.25 mm).

**Figure 2:**
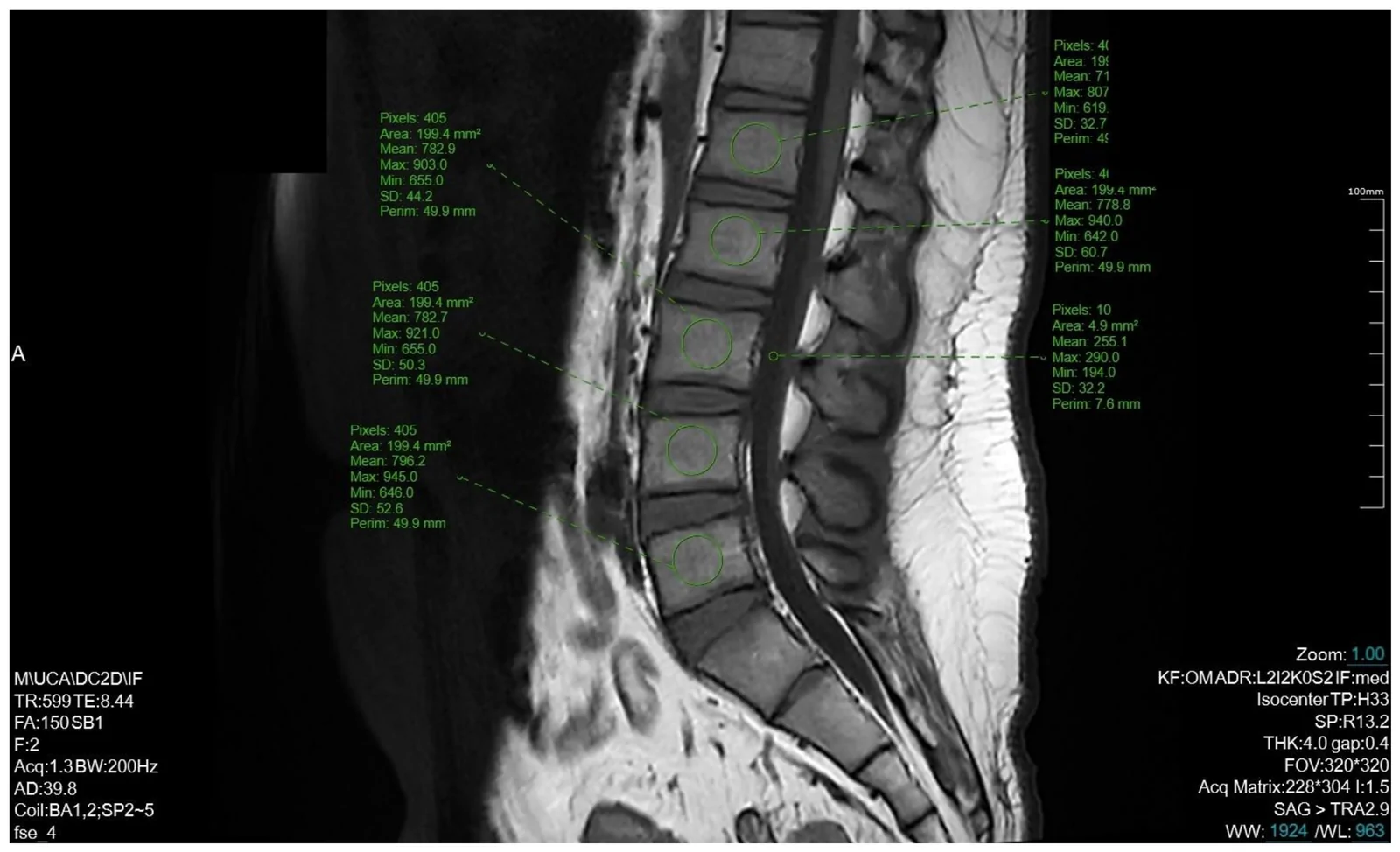
Representative VBQ measurement in a 21-year-old man with normal QCT-defined bone density. The image is a noncontrast T1-weighted midsagittal section of the lumbar spine acquired on a 1.5-T uMR 588 scanner (United Imaging Healthcare; repetition time, 599 ms; echo time, 8.44 ms; slice thickness, 4 mm; interslice gap, 0.4 mm). Equal-sized circular ROIs (199.4 mm^2^) were placed centrally in the L1-L4 vertebral bodies, yielding mean signal intensities of 712.4, 778.8, 782.9, and 796.2, respectively. A smaller ROI (4.9 mm^2^) was placed in the L3 cerebrospinal fluid, yielding a mean signal intensity of 255.1. The calculated VBQ(L1-L4) score was 3.01. QCT vBMD was 201.9 mg/cm^3^ at L1 and 196.8 mg/cm^3^ at L2 (mean, 199.35 mg/cm^3^), consistent with normal bone density.

VBQ measurements were performed on the noncontrast T1-weighted sagittal section closest to the true midsagittal plane. When spinal curvature prevented complete visualization of L1–L4, the nearest suitable parasagittal section was selected. Equal-sized circular ROIs were placed in the central trabecular marrow of the L1–L4 vertebral bodies, avoiding cortical bone, endplates, venous channels, focal lesions, and areas of abnormal signal. A smaller CSF reference ROI was placed in the ventral spinal canal at the L3 level on the same sagittal plane. The mean signal intensities of the four vertebral ROIs were averaged and normalized to the L3 CSF signal. Consistent with previously described principles [26], absolute MRI signal intensities were regarded as scanner-dependent values, whereas the normalized vertebral-to-CSF ratio represented the VBQ measure (Figure 2).

VBQ(L1–L4) = [SI(L1) + SI(L2) + SI(L3) + SI(L4)]/[4 × SI(CSF at L3)].

FF and R2* measurements were obtained from the corresponding quantitative sagittal MRI maps using anatomically matched L1–L4 vertebral ROIs. Source FF values were recorded in per mille (‰) and converted to percentages for reporting; R2* values were expressed in Hz. Participant-level FF and R2* measurements were calculated when at least three valid vertebral values were available. QCT examinations were performed using a uCT Atlas Pro CT scanner (United Imaging Healthcare, Shanghai, China). QCT trabecular volumetric bone mineral density (vBMD) was measured at L1 and L2 using Bone’s QCT software (version 2.0, Bone’s Technology (Shenzhen) Limited, Shenzhen, China). Regions of interest (ROIs) were centered within the vertebral bodies, avoiding cortical bone and focal lesions. The participant-level reference measurement was calculated as the mean of the two vertebrae. Normal bone density was defined as >120 mg/cm^3^, low bone mass as 80–120 mg/cm^3^, and osteoporosis as <80 mg/cm^3^.

All quantitative imaging measurements were independently performed by two readers using the same predefined ROI-placement protocol. Each reader completed the measurements independently, was unaware of the other reader’s measurements, and was blinded to the QCT reference-standard results when assessing the MRI-derived measurements. For the final dataset, the two readers’ measurements were averaged for each anatomical ROI, including the vertebral T1 signals and CSF signal; the primary VBQ value was then calculated from these averaged signals using the prespecified normalization formula. Participant-level FF, R2* values were derived from the corresponding averaged ROI measurements. Reader-specific VBQ scores were calculated separately to assess interobserver agreement and exploratory reader-specific diagnostic performance. This order of operations distinguishes the main VBQ measure from the arithmetic mean of two independently normalized reader-specific VBQ ratios.

### 2.3. Measurement Validity Criteria

Missing and nonnumeric entries were treated as unavailable. Individual FF values outside 0–1000‰ and R2* values outside 0–300 Hz were considered invalid and were excluded from their corresponding participant-level averages; at least three valid vertebral measurements were required. For the primary QCT-referenced analysis, both L1 and L2 vBMD measurements were required to fall within 0–350 mg/cm^3^. Participants with negative or grossly elevated QCT values, or with only a single evaluable QCT level, were excluded from this analysis. The possible influence of these exclusions on sample selection and diagnostic estimates was examined in additional exploratory analyses.

### 2.4. Statistical Analysis

Statistical analyses were performed using IBM SPSS Statistics for Windows, version 27.0 (IBM Corp., Armonk, NY, USA). Continuous variables are reported as mean ± standard deviation and categorical variables as number (percentage). Differences among QCT-defined bone-density groups were evaluated using Welch analysis of variance for continuous variables and the chi-square test for sex. For post hoc contrasts between the three QCT-defined groups, Games–Howell pairwise tests were used for age, VBQ, FF, R2*, and QCT vBMD; this procedure accounts for unequal group sizes and variances and adjusts for the three pairwise comparisons within each characteristic. Adjusted *p* values and simultaneous 95% confidence intervals for mean differences are reported in Supplementary Table S2. These are unadjusted descriptive comparisons with respect to clinical covariates, rather than causal effects. Pearson correlation coefficients were calculated with 95% confidence intervals derived using Fisher z transformation. Age- and sex-adjusted partial correlation between VBQ and QCT vBMD was estimated from linear-regression residuals. Cronbach’s alpha was used to assess internal consistency among L1–L4 T1 signal measurements, with bootstrap confidence intervals based on 5000 resamples.

Interobserver reliability was assessed using intraclass correlation coefficients (ICCs) from a two-way random-effects, absolute-agreement model. Both the single-measure ICC [ICC(2,1)] and average-measure ICC [ICC(2,2)] were calculated from the two reader-specific measurement series. The ICC(2,2) estimate characterizes the reliability of the mean of two reader-specific measurements; the primary VBQ analysis, as described above, instead normalized the averaged ROI signals. These closely related estimators are not mathematically identical. Confidence intervals were estimated using participant-level bootstrap resampling.

QCT-defined osteoporosis (<80 mg/cm^3^) was the binary outcome. Logistic regression models were fitted using (i) age and sex and (ii) age, sex, and VBQ. The incremental contribution of VBQ was evaluated using a likelihood-ratio test and comparison of the areas under the receiver operating characteristic curves (AUCs). To estimate optimism, model AUCs were corrected using 1000 bootstrap samples with model refitting. Standalone diagnostic performance was evaluated for VBQ, FF, and R2* in the same 234 participants; lower R2* values were treated as test-positive scores in this exploratory comparison. Case-stratified bootstrap resampling (2000 replicates) was used to estimate AUC intervals and paired differences. The cutoff maximizing the Youden index was identified in the original cohort and reselected in 1500 stratified bootstrap training samples; corresponding out-of-bag observations were used to assess threshold stability. Sensitivity, specificity, positive predictive value (PPV), negative predictive value (NPV), likelihood ratios, and accuracy were calculated at the observed cutoff, with Wilson 95% confidence intervals for key proportions. Age-stratified discrimination and comparisons between participants with and without valid QCT data were considered exploratory. All tests were two-sided, and *p* < 0.05 was considered statistically significant.

To examine whether exclusion of incomplete or numerically abnormal QCT measurements influenced the findings, two post hoc exploratory sensitivity analyses were performed. In the first analysis, the participant with only one available valid L1 or L2 QCT measurement was reinstated. In the second, participants were retained when at least one L1 or L2 measurement fell within the original numerical validity range of 0–350 mg/cm^3^, even if the other measurement was missing or outside that range. When two numerically valid measurements were available, their mean was used; otherwise, the single value was used as a provisional reference measurement. Out-of-range values were not imputed, truncated, or treated as valid. In both scenarios, the original osteoporosis definition of <80 mg/cm^3^ was applied. The age-and-sex and age-and-sex-plus-VBQ logistic models were refitted, and apparent AUCs and adjusted VBQ odds ratios with Wald 95% confidence intervals were recalculated. Sensitivity and specificity were also assessed using the original fixed VBQ cutoff of >3.7047 without selecting a new threshold. These analyses were intended to assess the robustness of the numerical exclusion rule, not to validate a single-vertebra QCT reference standard (Supplementary Table S1).

## 3. Results

### 3.1. Participant Characteristics and Data Flow

The primary MRI cohort comprised 278 participants (193 women [69.4%] and 85 men [30.6%]), with a mean age of 48.9 ± 21.1 years (range, 19–87 years). Of the 234 participants in the QCT-referenced cohort, 111 had normal bone density, 72 had low bone mass, and 51 had osteoporosis (Table 1). Participants included in the QCT-referenced analysis were younger than the 44 MRI-eligible participants without complete valid QCT data (mean age, 47.7 versus 55.5 years; *p* = 0.028). The proportions of women (69.2% versus 70.5%; Fisher *p* = 1.000) and mean VBQ scores (3.393 versus 3.448; *p* = 0.533) did not differ significantly between these groups.

### 3.2. Interobserver Reliability

Interobserver reliability was high for the participant-level quantitative imaging measurements. For VBQ, the single-measure ICC(2,1) was 0.851 (bootstrap 95% CI, 0.817–0.879), whereas the average-measure ICC(2,2), corresponding to the two-reader mean used in the primary analyses, was 0.919 (95% CI, 0.899–0.935). The average-measure ICC(2,2) was 0.947 for FF, 0.970 for R2*, and 0.968 for QCT vBMD. In an exploratory analysis, the VBQ AUC was 0.831 for reader 1 and 0.814 for reader 2, compared with 0.833 for the two-reader mean.

### 3.3. Internal Consistency

Cronbach’s alpha for the L1–L4 vertebral T1 signal measurements was 0.955 (bootstrap 95% CI, 0.928–0.976), indicating high internal consistency. This coefficient describes consistency across the four vertebral signal components; observer agreement was quantified separately by ICC.

### 3.4. Correlation Analyses

**Figure 3:**
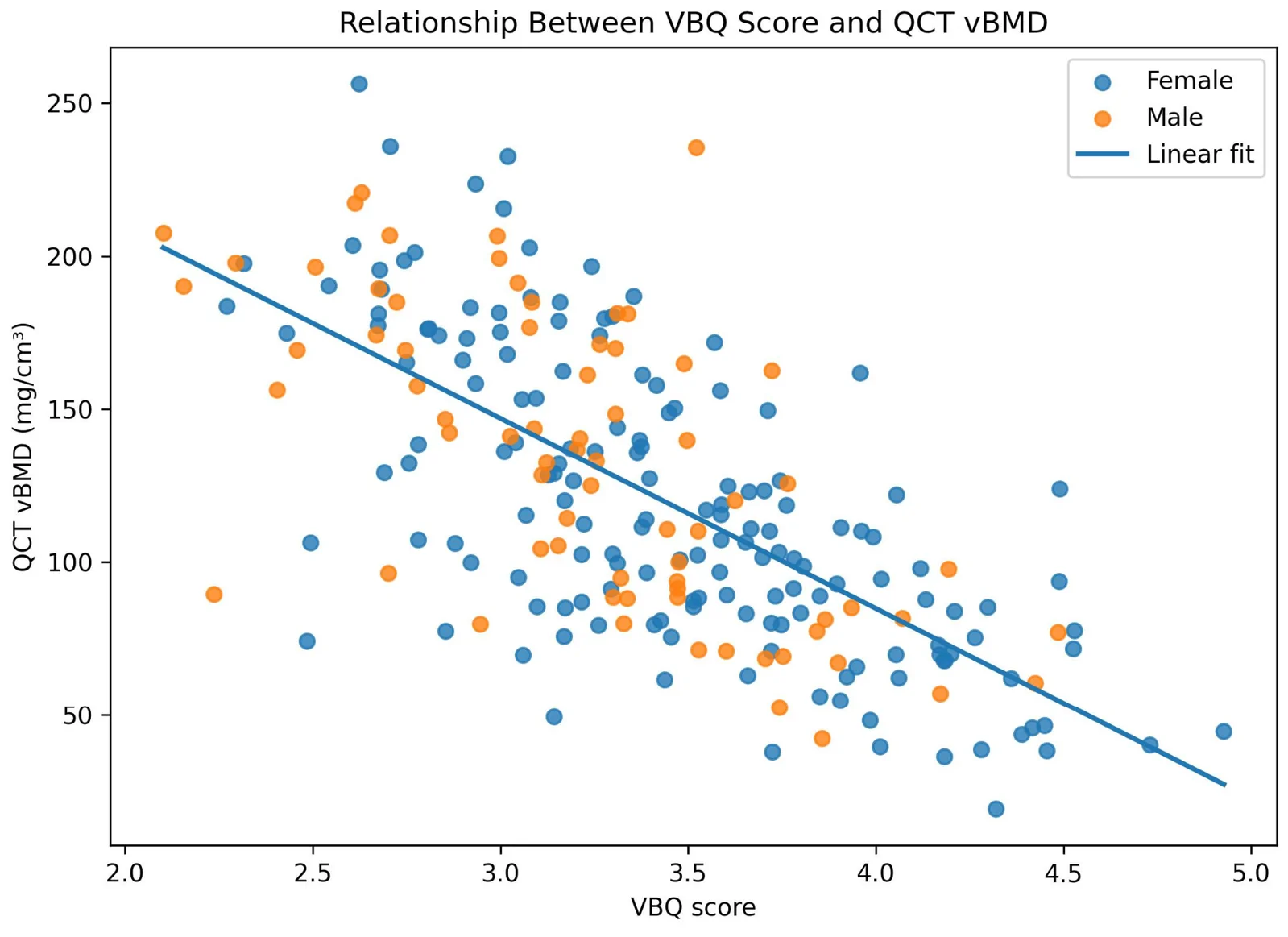
Relationship between the VBQ score and QCT-derived vertebral volumetric bone mineral density after exclusion of negative and grossly implausible QCT values.

**Figure 4:**
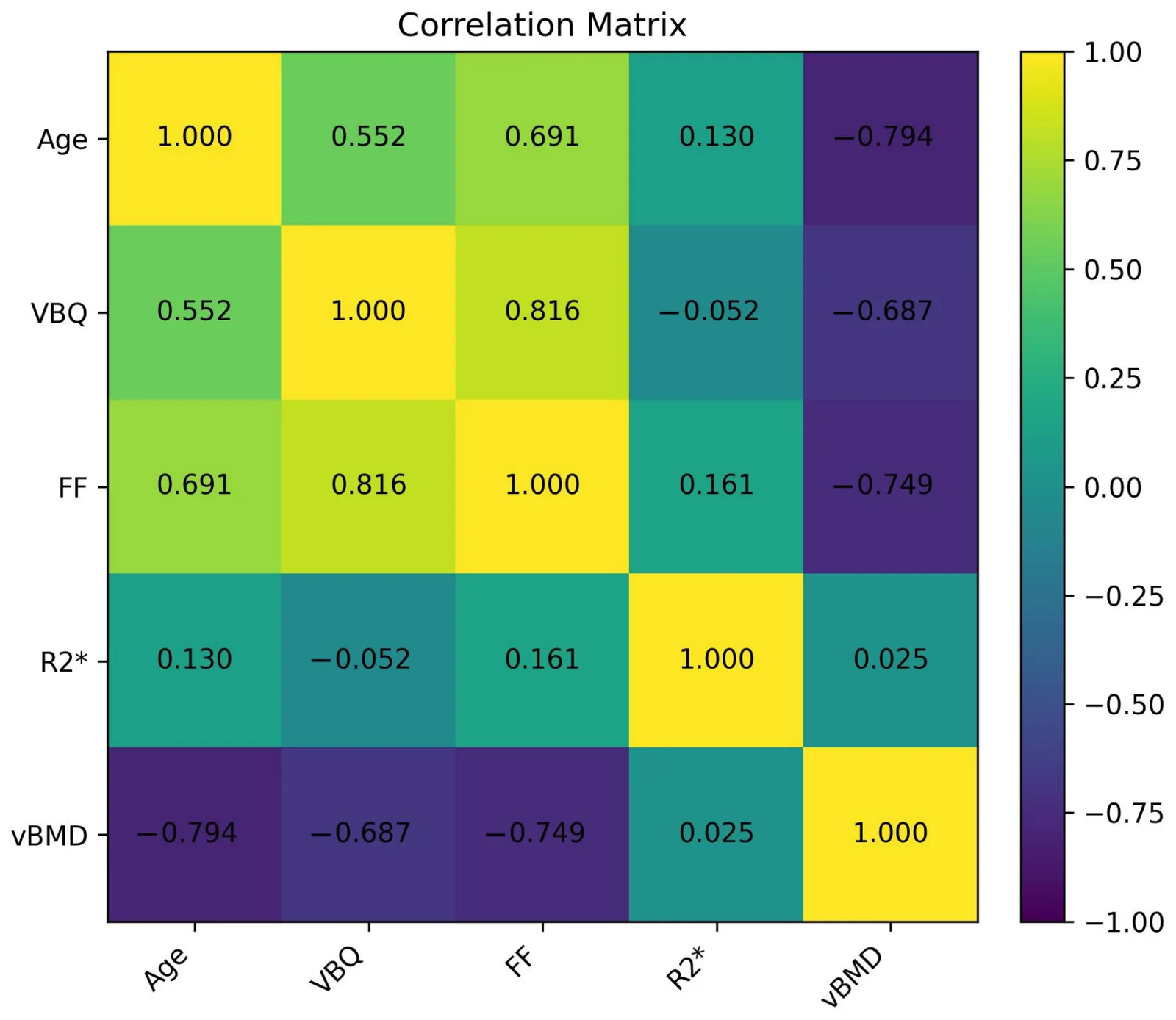
Correlation matrix for age, VBQ, FF, R2*, and QCT vBMD among 234 patients with valid QCT results. Non-QCT correlation pairs in Table 2 use the larger MRI cohort (*n* = 278), accounting for small differences in coefficients.

VBQ was strongly and positively correlated with FF and inversely correlated with QCT vBMD (r = −0.687; *p* < 0.001; Table 2). After adjustment for age and sex, the partial correlation between VBQ and QCT vBMD remained negative (r = −0.469; *p* < 0.001). FF was also strongly inversely correlated with vBMD, whereas R2* showed no meaningful association. The VBQ–vBMD relationship and the correlation matrix are shown in Figure 3 and Figure 4, respectively.

### 3.5. Between-Group Comparisons

**Figure 5:**
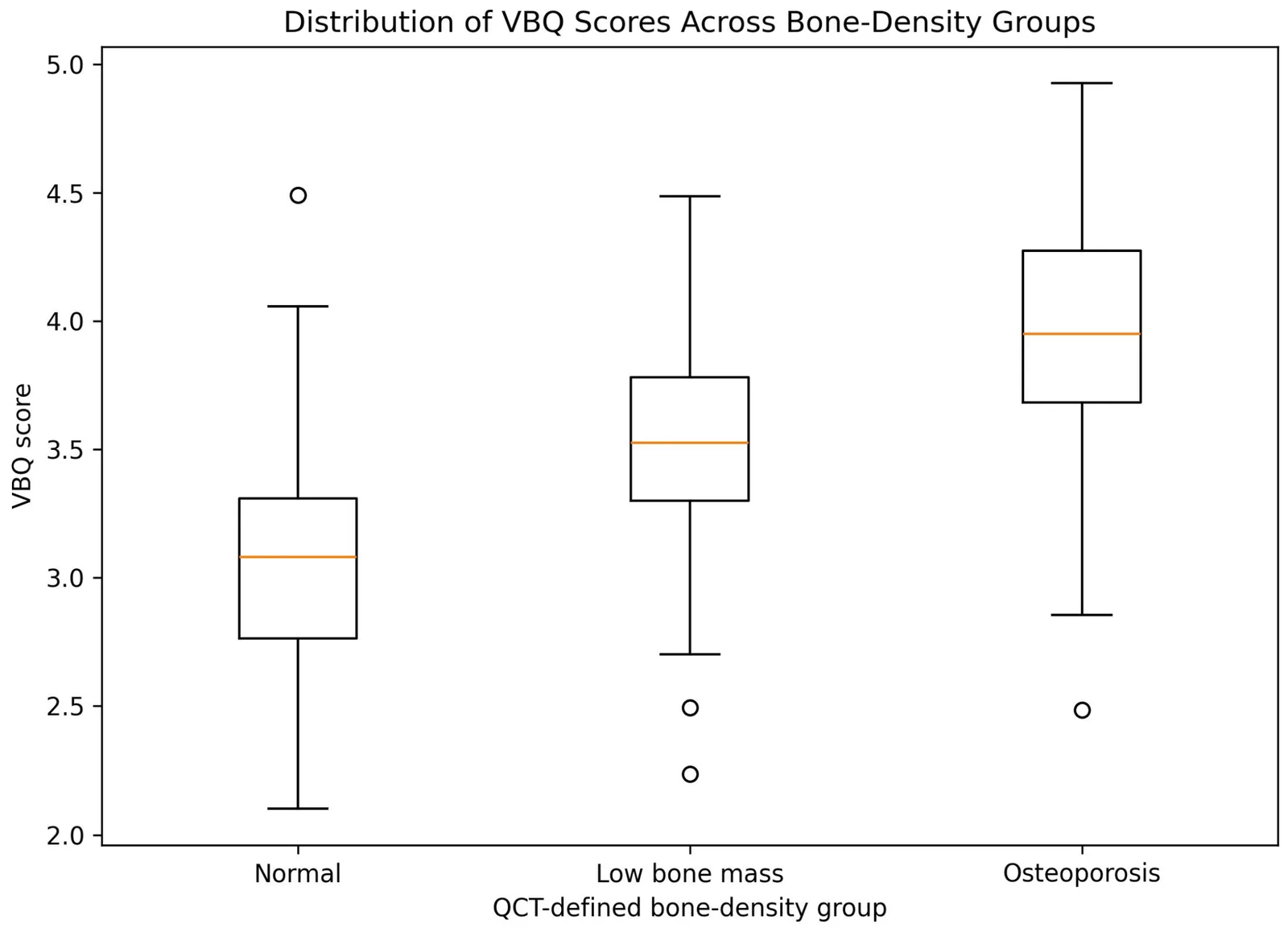
Distribution of VBQ scores across QCT-defined bone-density groups. Groups are ordered from normal bone density to low bone mass and osteoporosis.

VBQ increased progressively across the normal, low-bone-mass, and osteoporosis groups (3.071 ± 0.410, 3.522 ± 0.411, and 3.913 ± 0.508, respectively; *p* < 0.001). FF showed a similar stepwise pattern, whereas R2* did not differ significantly among groups (*p* = 0.122). Games–Howell post hoc tests, adjusted for the three pairwise comparisons within each continuous characteristic, showed differences among all three groups for age, VBQ, FF, and QCT vBMD (each pairwise adjusted *p* < 0.001). R2* did not differ significantly in any pairwise comparison (normal vs. low bone mass, *p* = 0.937; normal vs. osteoporosis, *p* = 0.269; low bone mass vs. osteoporosis, *p* = 0.153). These differences are descriptive, and differences in QCT vBMD are expected because the groups were defined using QCT thresholds. The group characteristics are presented in Table 1, the post hoc adjusted *p* values and simultaneous 95% confidence intervals in Supplementary Table S2, and the distribution of VBQ scores in Figure 5.

### 3.6. Adjusted Association with Osteoporosis

In multivariable logistic regression, each 1-unit increase in VBQ was associated with a 6.08-fold increase in the odds of QCT-defined osteoporosis after adjustment for age and sex (95% CI, 2.36–15.68; *p* < 0.001). Age was also independently associated with osteoporosis (OR per year, 1.13; 95% CI, 1.06–1.21; *p* < 0.001), whereas sex was not significant after adjustment (OR for women, 1.52; 95% CI, 0.60–3.90; *p* = 0.380). The apparent AUC increased from 0.847 for age and sex alone to 0.896 when VBQ was added (paired bootstrap AUC difference, 0.049; exploratory 95% interval, 0.003–0.093). The likelihood-ratio test also indicated an improvement in model fit (χ^2^ = 16.84, 1 df; *p* < 0.001). After bootstrap correction for optimism, the corresponding AUC estimates were 0.839 and 0.887. Detailed regression results are presented in Table 3.

### 3.7. Diagnostic Performance and Exploratory Threshold Selection

**Figure 6:**
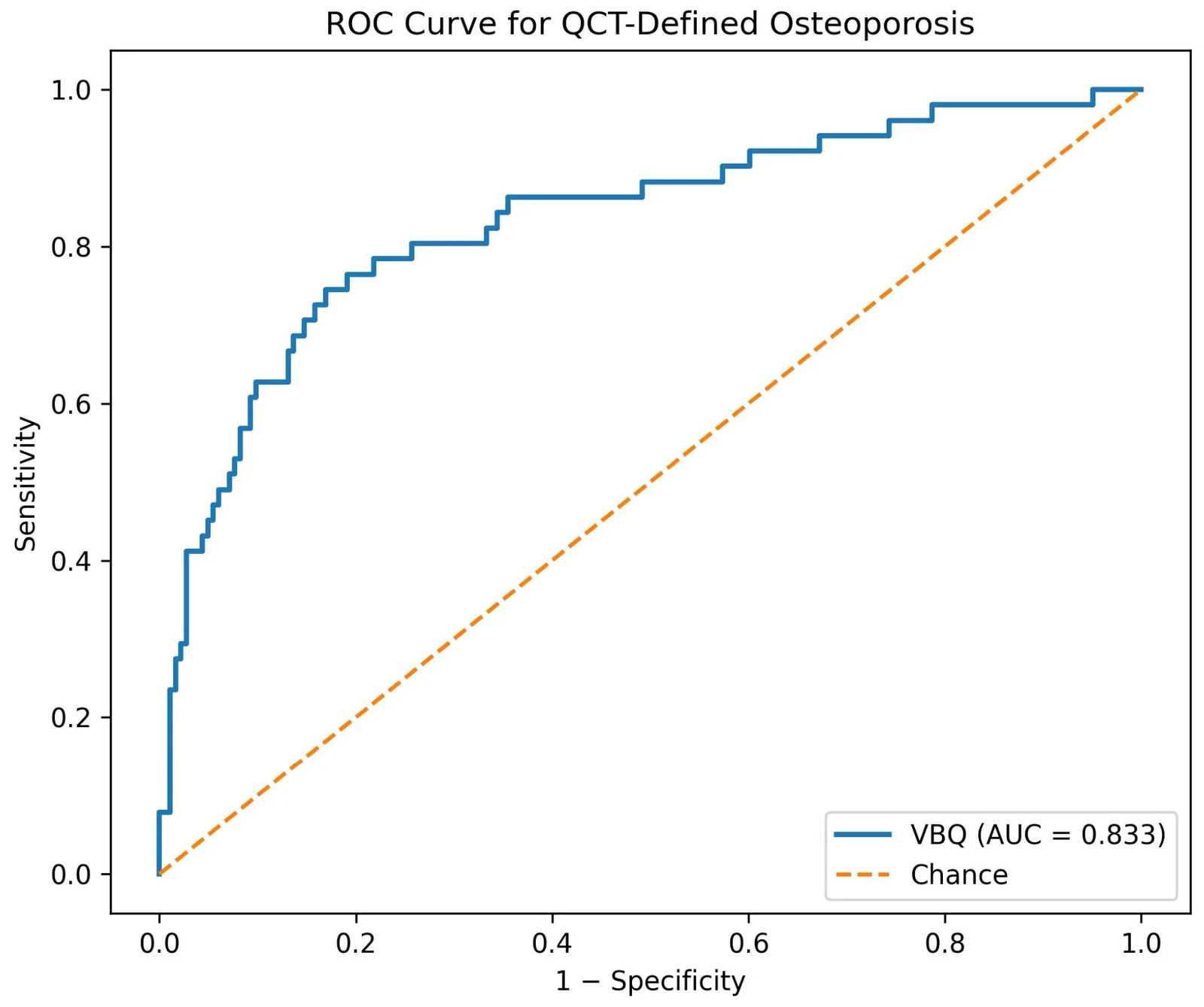
Receiver operating characteristic curve for the VBQ score in identifying QCT-defined osteoporosis.

The standalone AUC of VBQ for QCT-defined osteoporosis was 0.833 (2000-resample stratified-bootstrap 95% CI, 0.760–0.896). The cutoff maximizing the Youden index in the original cohort was VBQ >3.7047. At this threshold, sensitivity was 74.51% (38/51; Wilson 95% CI, 61.13–84.45%), specificity was 83.06% (152/183; 95% CI, 76.96–87.80%), PPV was 55.07% (38/69), and NPV was 92.12% (152/165). Thirteen of the 51 participants with osteoporosis were classified as test-negative. The corresponding ROC curve is shown in Figure 6, and the diagnostic characteristics of selected thresholds are summarized in Table 4.

In this cohort, the threshold of 3.5 provided higher sensitivity (80.39%) but lower specificity (71.04%), whereas 3.9 provided higher specificity (91.80%) at the expense of sensitivity (54.90%). These tradeoffs should be interpreted in the context of the clinical consequences of missed cases and false-positive referrals.

### 3.8. Additional Diagnostic Markers and Bootstrap Validation

**Figure 7:**
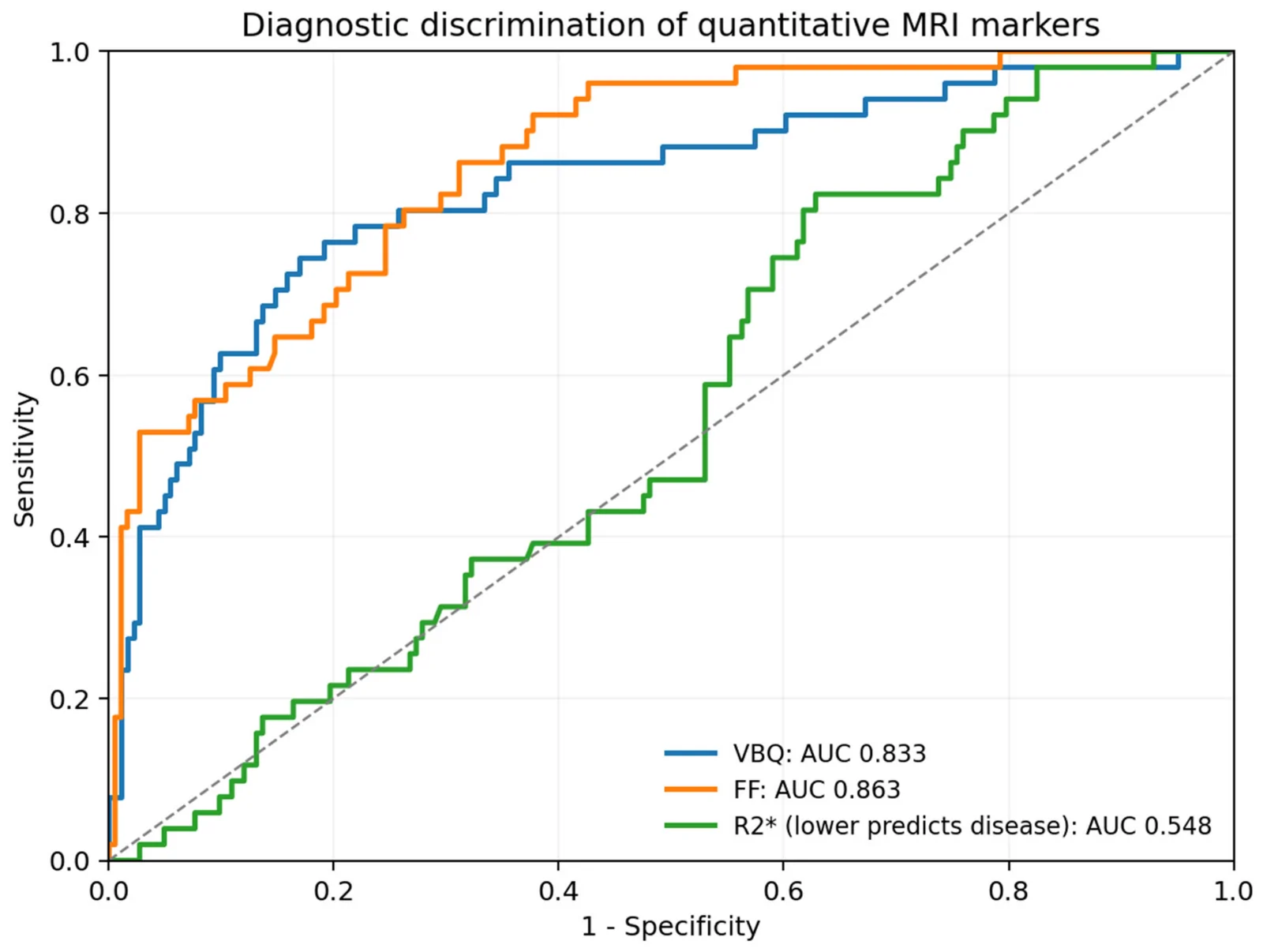
ROC curves comparing VBQ, FF, and R2* in the same QCT-referenced cohort (n = 234). R2* was evaluated exploratorily with lower values considered test-positive; all curves show in-sample discrimination.The gray dashed diagonal line represents the reference line of no discrimination (AUC = 0.50).

Within the same QCT-referenced cohort, the standalone AUC was 0.833 for VBQ, 0.863 for FF, and 0.548 for R2* when lower R2* values were considered test-positive. Although FF showed a numerically higher AUC than VBQ, the exploratory paired difference was 0.030 (bootstrap 95% interval, −0.025–0.088; *p* = 0.291), and the interval included zero.The AUC estimates for individual MRI-derived markers and the age- and sex-adjusted diagnostic models are summarized in Table 5. The receiver operating characteristic (ROC) curves for VBQ, FF, and R2* in the same QCT-referenced cohort are presented in Figure 7.

**Table 5:** AUC estimates for MRI markers and age/sex models.

Predictor	AUC	95% Bootstrap CI	Comments
VBQ	0.833	0.760–0.896	Standalone
FF (%)	0.863	0.807–0.913	Standalone
R2* (Hz)	0.548	0.468–0.627	Inverse direction; exploratory
Age + sex	0.847	0.790–0.897	Apparent
Age + sex + VBQ	0.896	0.847–0.935	Apparent

Abbreviations: AUC, area under the receiver operating characteristic curve; CI, confidence interval; VBQ, vertebral bone quality; FF, fat fraction; R2*, effective transverse relaxation rate. The 95% confidence intervals were estimated using bootstrap resampling. The AUCs for the age- and sex-based models are apparent (uncorrected) estimates. R2* was evaluated in the inverse direction as an exploratory analysis.

In 1500 stratified bootstrap samples, the median Youden-optimal VBQ cutoff was 3.7047, with 2.5th and 97.5th percentile values of 3.409 and 3.851. When the bootstrap-selected cutoffs were applied to out-of-bag observations, mean sensitivity was 70.36% and mean specificity was 81.38%. The apparent and optimism-corrected discrimination estimates for the regression models, together with the cutoff-stability results, are summarized in Table 6.

### 3.9. Age-Stratified and QCT-Availability Analyses

Among the 87 QCT-referenced participants aged 18–44 years, no cases of QCT-defined osteoporosis were observed; therefore, an age-specific AUC could not be calculated. In participants aged 45–59 years (*n* = 41, nine cases), VBQ had an AUC of 0.840, compared with 0.740 among those aged ≥60 years (*n* = 106, 42 cases). Participants without complete valid QCT results were older than those included in the reference-standard cohort, suggesting that the pooled diagnostic estimates may not apply equally to all patients undergoing lumbar MRI.

### 3.10. Exploratory Sensitivity Analysis of QCT Exclusions

Ten MRI-eligible participants had QCT records that did not satisfy the primary requirement for valid measurements at both L1 and L2. Of these, one had only one recorded vertebral value, six had a negative QCT value at one level, and three had at least one value >350 mg/cm^3^. In the latter group, two participants had values of 425 mg/cm^3^ at both levels; a third participant had one markedly elevated value and a numerically admissible value at the other level. Under the alternative rule requiring at least one numerically valid vertebral measurement, eight of the ten participants could be included: one with a single available value and seven with one in-range value paired with an invalid value. Two participants with neither vertebral value in the prespecified range remained excluded. The 34 participants without QCT results could not be classified under either alternative rule.

Reinstating only the participant with one available QCT level increased the sample to 235 participants, including 51 with osteoporosis. The adjusted VBQ odds ratio was 6.26 (95% CI, 2.43–16.16), and the standalone VBQ AUC remained 0.833. When all participants with at least one numerically valid QCT level were retained, the cohort comprised 242 participants, including 53 with osteoporosis. The standalone VBQ AUC was 0.838, compared with 0.833 in the primary analysis. The apparent AUCs were 0.839 versus 0.847 for the age-and-sex model and 0.892 versus 0.896 for the age-and-sex-plus-VBQ model, giving corresponding incremental AUCs of 0.053 and 0.049. The adjusted VBQ odds ratio was 7.18 (95% CI, 2.78–18.56), compared with 6.08 (95% CI, 2.36–15.68) in the original cohort. At the unchanged VBQ cutoff of >3.7047, sensitivity was 75.47% versus 74.51%, and specificity was 83.60% versus 83.06%. Thus, the main diagnostic estimates were broadly similar across the numerical exclusion rules. However, these analyses are exploratory because the retained single-vertebra QCT values had not been independently confirmed as technically valid reference-standard measurements (Supplementary Table S1).

## 4. Discussion

The principal findings were that VBQ showed a strong inverse association with QCT vBMD, high interobserver reliability, and good apparent discrimination for QCT-defined osteoporosis. Importantly, VBQ remained independently associated with osteoporosis after adjustment for age and sex and provided additional statistical discrimination beyond these demographic predictors. However, the cutoff derived in the present cohort varied across bootstrap samples and failed to identify a substantial proportion of patients with osteoporosis. These findings support the use of VBQ as an adjunctive opportunistic marker on routine lumbar MRI, rather than as a stand-alone diagnostic test.

The biological rationale for VBQ is that reduced bone density is often accompanied by increasing vertebral marrow adiposity, which raises marrow signal intensity on T1-weighted MRI [10,11,12,13,14]. The strong positive correlation between VBQ and FF in the present study is consistent with this mechanism. FF had a numerically higher AUC than VBQ (0.863 versus 0.833), although the paired bootstrap interval for the difference included zero. Direct FF quantification requires a dedicated quantitative MRI measurement, whereas VBQ can be calculated from routinely acquired T1-weighted images without an additional sequence [15,16,17,26]. R2* showed limited standalone discrimination in the exploratory direction assessed.

Published VBQ thresholds vary because of differences in MRI field strength, acquisition protocols, ROI methodology, study population, and reference standard [24,25,29]. The cutoff of 3.7047 in the present cohort was broadly consistent with previously reported 1.5-T values [25]. Nevertheless, it was derived and evaluated in the same patients, and the bootstrap-selected optimal values ranged from 3.409 to 3.851 between the 2.5th and 97.5th percentiles. Mean out-of-bag sensitivity was 70.36%, lower than the apparent sensitivity of 74.51%. These findings suggest that the numerical threshold is not yet sufficiently stable for general clinical application. Differences in the QCT criteria used to define osteoporosis across populations may introduce additional variability [31].

The adjusted analyses are clinically relevant because age differed substantially across QCT-defined groups. The age-and-sex model had an apparent AUC of 0.847, which increased to 0.896 after the inclusion of VBQ. This improvement supports an incremental association beyond basic demographic factors; however, improved discrimination and statistical fit do not necessarily establish clinical benefit. Diagnostic performance was lower among participants aged ≥60 years (AUC, 0.740), further emphasizing the influence of population characteristics. In addition, participants without complete valid QCT measurements were older than those included in the primary analysis, raising concerns about selection and partial verification bias. Any clinical use of VBQ should therefore remain complementary to DXA or QCT, fracture history, and established risk factors. In preoperative spine assessment, an elevated opportunistic VBQ score could prompt a formal bone-health workup before decisions about fixation or instrumentation, but the selection of screw trajectories and operative strategies requires assessment of local anatomy, bone quality, and other clinical factors. Because fixation trajectories and postoperative outcomes were not collected, no surgical risk-prediction or implant-guidance benefit can be inferred from this study [17,21,23].

### Limitations

This study has several limitations. First, the single-center retrospective design and nonrandom availability of QCT introduce selection and partial verification bias, particularly because patients lacking complete valid QCT measurements were older. Second, the VBQ threshold was derived and evaluated within the same cohort. Although bootstrap resampling estimated optimism and threshold variability, independent multicenter validation was not available. Third, all examinations used a single 1.5-T MRI platform, and the cutoff may not transfer to other scanners or protocols. Fourth, diagnostic discrimination varied across age groups, and no osteoporosis cases were present among QCT-evaluable participants aged 18–44 years. Fifth, the VBQ score was calculated from L1–L4, whereas QCT vBMD was measured at L1–L2, resulting in an anatomical mismatch. The de-identified analytic workbook did not contain coded individual MRI indication categories, preventing a reliable frequency breakdown of presenting symptoms or specific spinal diagnoses in this revision. The available data likewise did not permit independent confirmation of the QCT device-specific calibration logs or a uniform absolute ROI area across all patients. Finally, potentially relevant clinical and marrow-related confounders were not completely characterized, and the study did not examine longitudinal fracture outcomes or the consequences of VBQ-guided clinical decisions.

The exploratory QCT-exclusion sensitivity analyses provided broadly similar diagnostic estimates when eight additional participants were included using a single numerically admissible vertebral measurement. However, a value within the numerical range cannot be assumed to be technically reliable when the paired QCT result is negative or markedly discordant. The original QCT images and calibration or quality-control records should be reviewed before these provisional classifications are interpreted clinically. Furthermore, a single-vertebra measurement is not equivalent to the predefined two-vertebra reference standard. The alternative analyses also could not address potential verification bias in the 34 participants who had no QCT measurements. Therefore, these findings indicate limited robustness to changes in the numerical exclusion rule but do not demonstrate that selection bias has been eliminated.

Future studies should use prospective, representative cohorts with systematic QCT or DXA reference measurements, standardized imaging protocols, and prespecified criteria for handling incomplete or abnormal values. Multicenter external validation is needed to evaluate rounded, protocol-specific VBQ thresholds across scanners and age groups. Prospective studies should also determine whether VBQ improves clinical decision-making beyond established densitometric and risk-based assessments, rather than relying solely on improvements in AUC.

## 5. Conclusions

The MRI-based VBQ score showed a strong inverse association with QCT vBMD, high interobserver reliability for the two-reader average, and incremental apparent discrimination for QCT-defined osteoporosis beyond age and sex. The exploratory 1.5-T threshold of approximately 3.7 varied across bootstrap samples and failed to identify approximately one-quarter of patients with osteoporosis. VBQ may therefore provide an adjunctive marker for opportunistic case finding on routine lumbar MRI, but it should not be used alone to exclude osteoporosis. Independent multicenter validation is required before the threshold is generalized.

## Figures and Tables

**Table 1:** Characteristics of the QCT-referenced cohort.

Characteristic	Normal (*n* = 111)	Low Bone Mass (*n* = 72)	Osteoporosis (*n* = 51)	*p* Value
Age, years	30.9 ± 17.1	60.3 ± 9.8	66.4 ± 6.8	<0.001
Women, *n* (%)	70 (63.1)	53 (73.6)	39 (76.5)	0.143
VBQ score	3.071 ± 0.410	3.522 ± 0.411	3.913 ± 0.508	<0.001
FF, %	42.37 ± 10.24	54.21 ± 6.81	61.69 ± 8.17	<0.001
R2*, Hz	73.49 ± 19.23	74.40 ± 16.49	69.79 ± 10.86	0.122
QCT vBMD, mg/cm^3^	166.66 ± 30.42	97.89 ± 10.98	61.53 ± 14.85	<0.001

Values are mean ± standard deviation unless otherwise indicated. *p* values for continuous variables were obtained using Welch analysis of variance; the sex distribution was compared using the chi-square test. Post hoc Games–Howell comparisons (multiple-comparison-adjusted within each continuous characteristic) are shown in Supplementary Table S2.

**Table 2:** Pearson correlations among VBQ, FF, R2*, and QCT vBMD.

Variables	*n*	r	95% CI	*p* Value
VBQ vs. FF	278	0.806	0.761 to 0.844	<0.001
VBQ vs. R2*	278	−0.059	−0.175 to 0.059	0.327
VBQ vs. vBMD	234	−0.687	−0.749 to −0.612	<0.001
FF vs. R2*	278	0.155	0.038 to 0.268	0.010
FF vs. vBMD	234	−0.749	−0.800 to −0.687	<0.001
R2* vs. vBMD	234	0.025	−0.104 to 0.152	0.708

**Table 3:** Adjusted logistic regression for QCT-defined osteoporosis.

Predictor	β	SE	Adjusted OR (95% CI)	*p* Value
VBQ per 1 unit	1.805	0.483	6.08 (2.36–15.68)	<0.001
Age per year	0.125	0.035	1.13 (1.06–1.21)	<0.001
Female sex	0.421	0.479	1.52 (0.60–3.90)	0.380

OR, odds ratio; CI, confidence interval; age + sex apparent AUC = 0.847; age + sex + VBQ apparent AUC = 0.896; bootstrap optimism-adjusted AUCs = 0.839 and 0.887.

**Table 4:** Diagnostic performance at selected VBQ thresholds.

Threshold	Sensitivity, %	Specificity, %	PPV, %	NPV, %	LR+	LR−	Accuracy, %
3.5	80.39	71.04	43.62	92.86	2.78	0.28	73.08
3.7047	74.51	83.06	55.07	92.12	4.40	0.31	81.20
3.9	54.90	91.80	65.12	87.96	6.70	0.49	83.76

PPV, positive predictive value; NPV, negative predictive value; LR+, positive likelihood ratio; LR−, negative likelihood ratio. At the data-derived >3.7047 threshold, TP = 38, FP = 31, TN = 152, and FN = 13. Metrics were estimated in the same patients used to derive this threshold.

**Table 6:** Internal validation and cutoff stability.

Resampling Statistic	Estimate	Notes
Median VBQ cutoff	3.7047	1500 stratified bootstrap samples
Cutoff percentiles, 2.5th–97.5th	3.409–3.851	Sampling variability; not external limits
Out-of-bag sensitivity (mean)	70.36%	Selected cutoff per sample
Out-of-bag specificity (mean)	81.38%	Selected cutoff per sample
Age + sex optimism-corrected AUC	0.839	1000 bootstrap refits
Age + sex + VBQ corrected AUC	0.887	1000 bootstrap refits

## Data Availability

The data presented in this study are available from the corresponding author upon reasonable request, subject to participant privacy and applicable ethical requirements for individual-level clinical and imaging data.

## Supplementary Materials

The following supporting information can be downloaded at https://www.mdpi.com/article/10.3390/jcm15218752/s1. Table S1. Sensitivity of diagnostic estimates to alternative QCT data-validity rules; Table S2. Games–Howell post hoc pairwise comparisons among QCT-defined bone-density groups.
