# Supplementary material for: MRI-Based Vertebral Bone Quality Score for QCT-Defined Osteoporosis: Incremental Diagnostic Value and an Exploratory 1.5-T Threshold in a Retrospective Study
Source: J Clin Med. 2026 Nov 9;15(21):8752. doi: 10.3390/jcm15218752 (PMC13764616; doi:10.3390/jcm15218752)
Supplement: Supplementary file 1 [file jcm-15-08752-s001.zip › jcm-4644365-supplementary.pdf]

Supplementary Materials

MRI-Based Vertebral Bone Quality Score for QCT-Defined Osteoporosis: Incremental Diagnostic Value and an Exploratory 1.5-T Threshold in a Retrospective Study

Manuscript ID: jcm-4644365

Table S1. Sensitivity of diagnostic estimates to alternative QCT data-validity rules.

Analysis rule	Total n	Cases n	VBQ AUC	Age + sex AUC	Age + sex + VBQ AUC	ΔAUC	Adjusted VBQ OR (95% CI)	Sensitivity (%)	Specificity (%)
Primary analysis: both L1/L2 valid	234	51	0.833	0.847	0.896	0.049	6.08 (2.36–15.68)	74.51	83.06
Single available valid QCT value recovered	235	51	0.833	0.845	0.896	0.051	6.26 (2.43–16.16)	74.51	83.15
At least one numerically valid QCT level	242	53	0.838	0.839	0.892	0.053	7.18 (2.78–18.56)	75.47	83.60

Note 1. Data source and reference definition: Analyses were performed from the de-identified original study workbook (averaged measurements from two readers). Osteoporosis was defined as QCT volumetric bone mineral density <80 mg/cm³. The primary analysis required valid L1 and L2 values (each 0–350 mg/cm³) and used their average.

Note 2. Alternative-rule definitions: The second row additionally included the one participant with only one available valid QCT level. The third row additionally retained seven participants with one valid L1/L2 measurement despite an invalid other measurement. For those with a single valid value, that value provisionally replaced the two-level mean; no negative, >350 mg/cm³, or missing values were treated as valid. Two individuals with both vertebral values outside 0–350 mg/cm³ remained excluded.

Note 3. Statistical definitions: VBQ AUC is the apparent ROC AUC for the VBQ score alone. ΔAUC is the apparent AUC of (age + sex + VBQ) minus that of (age + sex), within each analytic cohort. Adjusted OR represents the odds ratio for one unit higher VBQ from logistic regression adjusted for age and sex; 95% confidence intervals were calculated using the Wald method. Sensitivity and specificity used the original fixed VBQ cutoff >3.7047, without threshold re-estimation.

Note 4. Interpretation and restrictions: These post hoc exploratory analyses describe sensitivity to numerical exclusion criteria only. The extra single-level QCT measurements have not been independently confirmed by imaging review or calibration-quality assurance, and a single-level reference is not interchangeable with the originally specified two-level mean. Thirty-four MRI participants without QCT cannot be classified in any of these scenarios. Results do not eliminate selection or partial-verification bias.

Table S2. Games–Howell post hoc pairwise comparisons among QCT-defined bone-density groups.

Characteristic	Pairwise comparison	Mean difference (simultaneous 95% CI)	Adjusted P
Age, years	Normal − Low bone mass	-29.44 (-34.15 to -24.74)	<0.001
Age, years	Normal − Osteoporosis	-35.52 (-39.98 to -31.06)	<0.001
Age, years	Low bone mass − Osteoporosis	-6.08 (-9.63 to -2.53)	<0.001
VBQ score	Normal − Low bone mass	-0.45 (-0.60 to -0.30)	<0.001
VBQ score	Normal − Osteoporosis	-0.84 (-1.04 to -0.65)	<0.001
VBQ score	Low bone mass − Osteoporosis	-0.39 (-0.60 to -0.19)	<0.001
FF, %	Normal − Low bone mass	-11.84 (-14.82 to -8.86)	<0.001
FF, %	Normal − Osteoporosis	-19.32 (-22.89 to -15.76)	<0.001
FF, %	Low bone mass − Osteoporosis	-7.48 (-10.81 to -4.16)	<0.001
R2*, Hz	Normal − Low bone mass	-0.91 (-7.22 to 5.39)	0.937
R2*, Hz	Normal − Osteoporosis	3.69 (-1.93 to 9.31)	0.269
R2*, Hz	Low bone mass − Osteoporosis	4.61 (-1.25 to 10.46)	0.153
QCT vBMD, mg/cm³	Normal − Low bone mass	68.77 (61.28 to 76.26)	<0.001
QCT vBMD, mg/cm³	Normal − Osteoporosis	105.14 (96.72 to 113.55)	<0.001
QCT vBMD, mg/cm³	Low bone mass − Osteoporosis	36.37 (30.53 to 42.21)	<0.001

Mean differences are calculated as the first group minus the second group. P values account for the three pairwise comparisons within each characteristic using the Games–Howell studentized-range procedure; confidence intervals are simultaneous 95% Games–Howell intervals. These comparisons were not adjusted across the five different characteristics. QCT vBMD differences are descriptive because QCT is the basis of group assignment.
